# Supplementary material for: Virulence Determinants of Colistin-Resistant K. pneumoniae High-Risk Clones
Source: Biology (Basel). 2021 May 14;10(5):436. doi: 10.3390/biology10050436 (PMC8155863; doi:10.3390/biology10050436)
Supplement: Supplementary file 1 [file biology-10-00436-s001.zip › Virulence Determinants of Colistin-Resistant K.pneumoniae High-Risk Clones.pdf]

Virulence Determinants of Colistin-Resistant *K.pneumoniae* High-Risk Clones

May 11, 2021

Summary table

```
##                    Total             ST101              ST395              Other STs          p (ST101) p (ST395)
## 30-day mortality " 72/142 (50.7%)" "46/80 (57.5%)"   "12/16 (75.0%)"   "14/46 (30.4%)" "0.005"   "0.003"
## Bacteremia       " 61/142 (43.0%)" "33/80 (41.2%)"   "10/16 (62.5%)"   "18/46 (39.1%)" "0.852"   "0.147"
## Carbapenem res.  "135/142 (95.1%)" "78/80 (97.5%)"   "16/16 (100.0%)"  "41/46 (89.1%)" "0.098"   "0.315"
## Colistin exp.    " 69/142 (48.6%)" "36/80 (45.0%)"   " 5/16 (31.2%)"   "28/46 (60.9%)" "0.098"   "0.048"
## Colistin tar.    " 55/142 (38.7%)" "31/80 (38.8%)"   " 4/16 (25.0%)"   "20/46 (43.5%)" "0.707"   "0.242"
## Comorbidity      " 49/142 (34.5%)" "27/80 (33.8%)"   " 4/16 (25.0%)"   "18/46 (39.1%)" "0.567"   "0.375"
## Emp. carbapenem  " 68/141 (48.2%)" "39/80 (48.8%)"   " 3/15 (20.0%)"   "26/46 (56.5%)" "0.461"   "0.018"
## Emp. colistin    " 42/141 (29.8%)" "20/80 (25.0%)"   " 1/16 (6.2%)"    "21/45 (46.7%)" "0.017"   "0.005"
## FimH             "136/142 (95.8%)" "79/80 (98.8%)"   "16/16 (100.0%)"  "41/46 (89.1%)" "0.024"   "0.315"
## FyuA             "138/142 (97.2%)" "79/80 (98.8%)"   "15/16 (93.8%)"   "44/46 (95.7%)" "0.553"   "1.000"
## Gender           " 60/142 (42.3%)" "34/80 (42.5%)"   " 7/16 (43.8%)"   "19/46 (41.3%)" "1.000"   "1.000"
## ICU              "119/141 (84.4%)" "68/79 (86.1%)"   "15/16 (93.8%)"   "36/46 (78.3%)" "0.323"   "0.261"
## IutA             " 10/142 (7.0%)"  " 4/80 (5.0%)"    " 1/16 (6.2%)"    " 5/46 (10.9%)" "0.285"   "1.000"
## K2Wzy            " 60/142 (42.3%)" "31/80 (38.8%)"   " 9/16 (56.2%)"   "20/46 (43.5%)" "0.707"   "0.401"
## Kfu              "121/142 (85.2%)" "79/80 (98.8%)"   "15/16 (93.8%)"   "27/46 (58.7%)" "0.000"   "0.012"
## KPC              "  2/142 (1.4%)"  " 0/80 (0.0%)"    " 0/16 (0.0%)"    " 2/46 (4.3%)"  "0.131"   "1.000"
## MagA             " 10/142 (7.0%)"  " 6/80 (7.5%)"    " 0/16 (0.0%)"    " 4/46 (8.7%)"  "1.000"   "0.565"
## Mortality        " 95/142 (66.9%)" "61/80 (76.2%)"   "12/16 (75.0%)"   "22/46 (47.8%)" "0.002"   "0.082"
## mrkD             "136/142 (95.8%)" "79/80 (98.8%)"   "15/16 (93.8%)"   "42/46 (91.3%)" "0.059"   "1.000"
## NDM-1            " 31/142 (21.8%)" " 3/80 (3.8%)"    "14/16 (87.5%)"   "14/46 (30.4%)" "0.000"   "0.000"
## Operation        " 73/142 (51.4%)" "40/80 (50.0%)"   " 8/16 (50.0%)"   "25/46 (54.3%)" "0.712"   "0.780"
## OXA-48           "116/142 (81.7%)" "76/80 (95.0%)"   " 5/16 (31.2%)"   "35/46 (76.1%)" "0.003"   "0.002"
## RmpA             "116/142 (81.7%)" "71/80 (88.8%)"   "14/16 (87.5%)"   "31/46 (67.4%)" "0.005"   "0.194"
## VAP              " 67/142 (47.2%)" "44/80 (55.0%)"   " 9/16 (56.2%)"   "14/46 (30.4%)" "0.009"   "0.079"
## WabG             "141/142 (99.3%)" "80/80 (100.0%)"  "16/16 (100.0%)"  "45/46 (97.8%)" "0.365"   "1.000"
## ybtS             "121/142 (85.2%)" "79/80 (98.8%)"   "15/16 (93.8%)"   "27/46 (58.7%)" "0.000"   "0.012"
```

Heatmap analysis

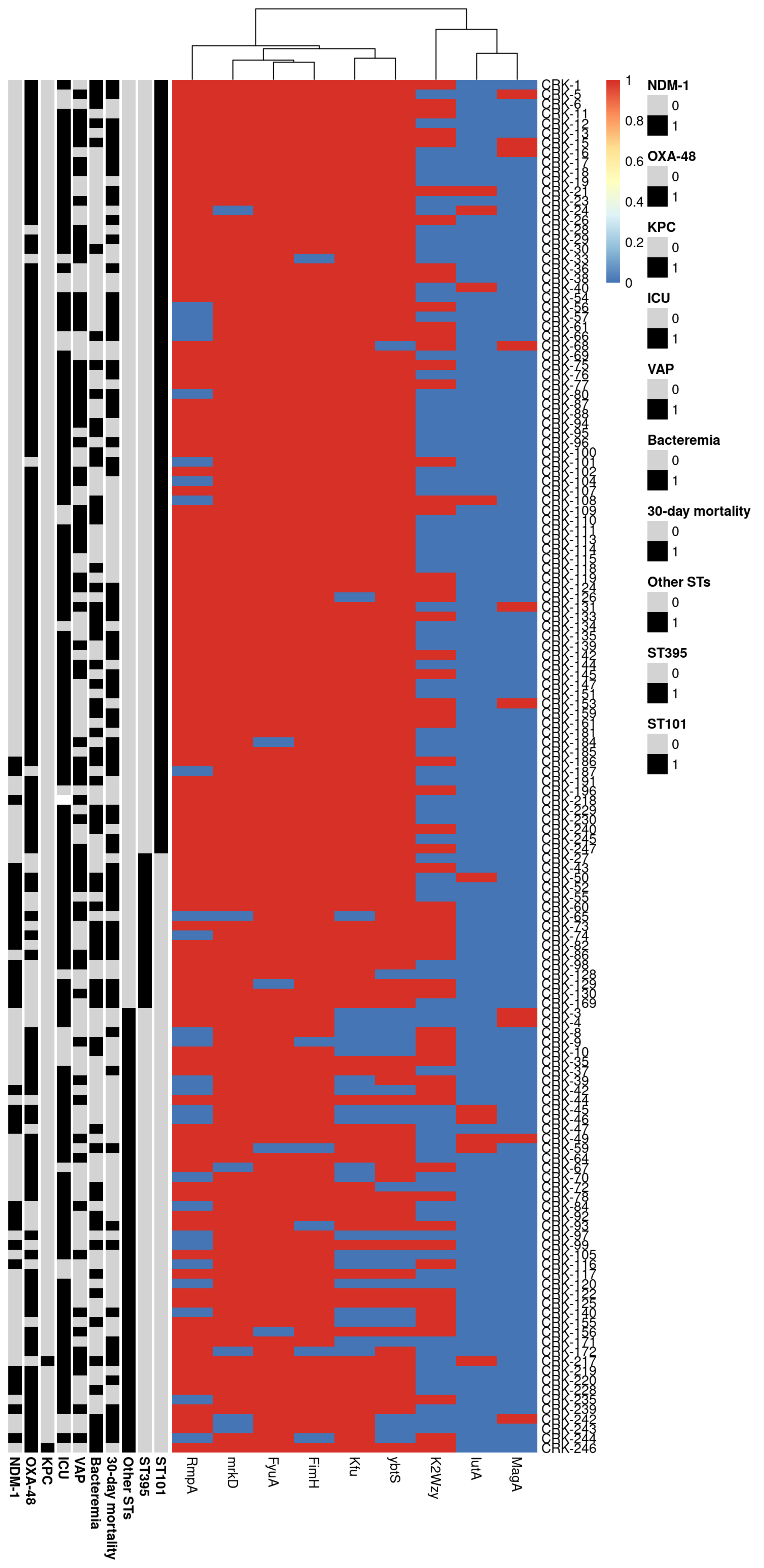

Minimum spanning tree

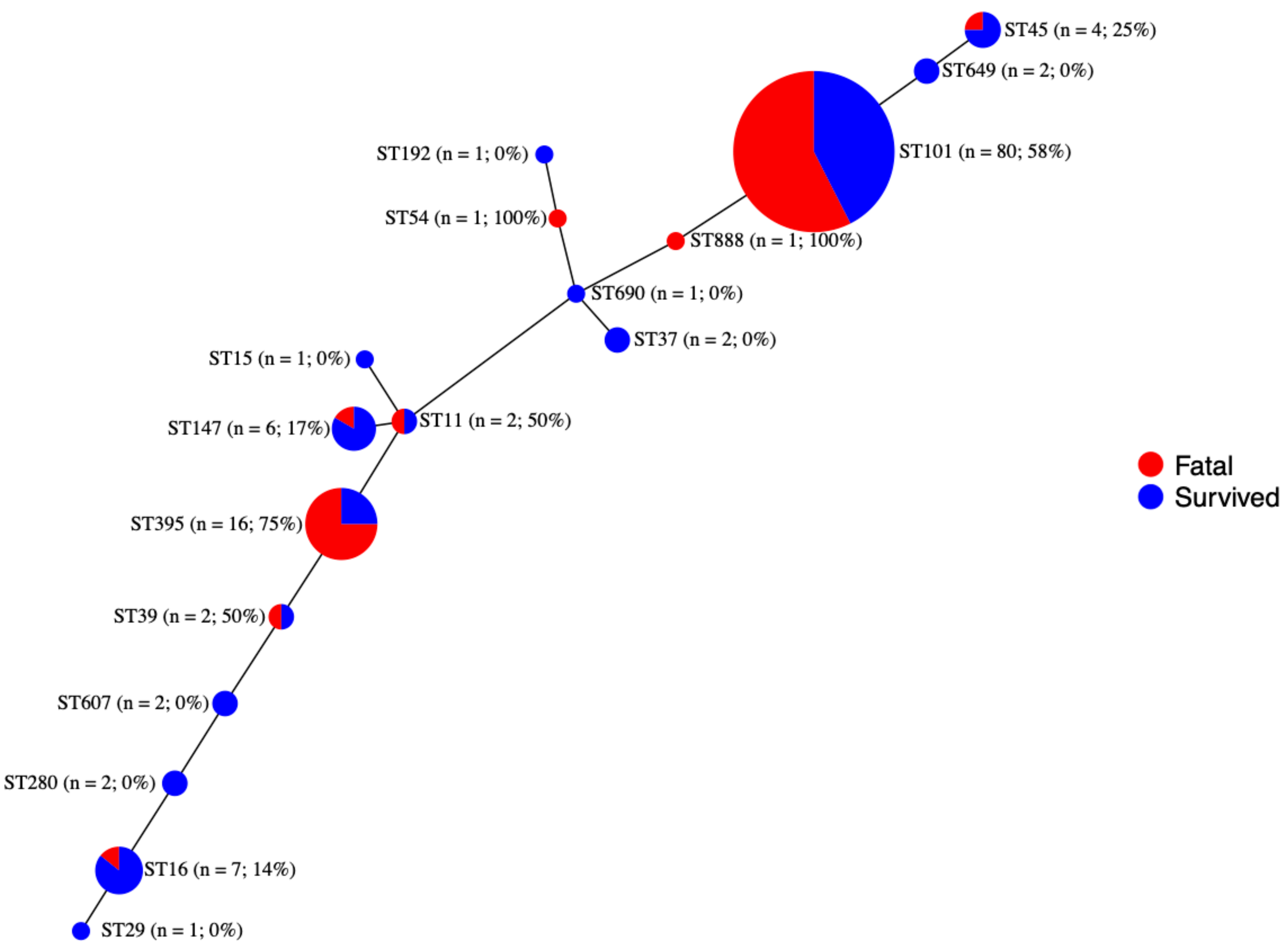

Fisher's exact test between 30-day mortality & other covariates

```
##            OR    CI            p-value
## Bacteremia "1.6" "0.78-3.31"   "0.179"
## FimH       "0.5" "0.04-3.64"   "0.681"
## FyuA       "0.3" "0.01-4.30"   "0.620"
## ICU        "4.3" "1.42-16.04"  "0.005"
## IutA       "0.6" "0.12-2.79"   "0.529"
## K2Wzy      "1.3" "0.66-2.78"   "0.401"
## Kfu        "3.9" "1.27-14.63"  "0.009"
## KPC        "1.0" "0.01-77.33"  "1.000"
## MagA       "1.0" "0.21-4.43"   "1.000"
## mrkD       "1.0" "0.13-7.96"   "1.000"
## NDM-1      "1.5" "0.61-3.58"   "0.419"
## OXA-48     "1.0" "0.40-2.66"   "1.000"
## RmpA       "1.2" "0.49-3.23"   "0.668"
## ST101      "1.9" "0.91-3.88"   "0.090"
## ST395      "3.3" "0.93-14.68"  "0.061"
## VAP        "1.8" "0.86-3.64"   "0.096"
## ybtS       "3.0" "1.01-10.02"  "0.034"
```

Multivariate analysis to predict 30-day mortality status

```
##       OR     CI            p-value
## ICU   "7.9"  "1.43-55.98"  "0.024"
## Kfu   "27.0" "5.67-179.65" "0.000"
## ST101 "17.2" "2.45-350.40" "0.014"
## ST395 "2.7"  "0.33-58.50"  "0.401"
## VAP   "3.2"  "0.60-20.74"  "0.192"
## ybtS  "0.7"  "0.13-3.17"   "0.624"
```

Multivariate analysis to predict ST101 status

```
##                  OR     CI            p-value
## 30-day mortality "1.2"  "0.17-6.12"   "0.860"
## FimH             "3.1"  "0.18-100.25" "0.464"
## Kfu              "20.3" "2.17-484.56" "0.018"
## Mortality        "3.4"  "0.71-22.83"  "0.156"
## mrkD             "4.3"  "0.24-130.73" "0.331"
## NDM-1            "0.0"  "0.01-0.15"   "0.000"
## OXA-48           "5.0"  "1.04-26.48"  "0.045"
## RmpA             "0.7"  "0.11-3.53"   "0.688"
## VAP              "1.8"  "0.61-5.64"   "0.292"
## ybtS             "9.9"  "1.08-228.71" "0.067"
```

Multivariate analysis to predict ST395 status

```
##                  OR          CI           p-value
## 30-day mortality "83887477.7" "0.00-NA"    "0.993"
## Kfu              "0.6"        "0.05-17.86" "0.717"
## Mortality        "0.0"        "0.00-Inf"   "0.993"
## OXA-48           "0.0"        "0.01-0.15"  "0.000"
## VAP              "1.9"        "0.52-8.08"  "0.336"
## ybtS             "1.4"        "0.11-43.51" "0.808"
```

Fisher's exact test between VAP & other covariates

```
##       OR    CI           p-value
## FimH  "0.4" "0.04-3.14"  "0.421"
## Kfu   "2.5" "0.85-8.50"  "0.096"
## RmpA  "1.1" "0.41-2.72"  "1.000"
## ST101 "2.1" "1.00-4.33"  "0.042"
## ST395 "1.5" "0.47-5.07"  "0.596"
## ybtS  "4.6" "1.38-19.76" "0.008"
```
